# *Poecilia picta*, a Close Relative to the Guppy, Exhibits Red Male Coloration Polymorphism: A System for Phylogenetic Comparisons

**DOI:** 10.1371/journal.pone.0142089

**Published:** 2015-11-03

**Authors:** Anna K. Lindholm, Ben Sandkam, Kristina Pohl, Felix Breden

**Affiliations:** 1 Institute for Evolutionary Biology and Environmental Studies, University of Zurich, Zurich, Switzerland; 2 Department of Biological Sciences, Simon Fraser University, Burnaby, British Columbia, Canada; University of Lausanne, SWITZERLAND

## Abstract

Studies on the evolution of female preference and male color polymorphism frequently focus on single species since traits and preferences are thought to co-evolve. The guppy, *Poecilia reticulata*, has long been a premier model for such studies because female preferences and orange coloration are well known to covary, especially in upstream/downstream pairs of populations. However, focused single species studies lack the explanatory power of the comparative method, which requires detailed knowledge of multiple species with known evolutionary relationships. Here we describe a red color polymorphism in *Poecilia picta*, a close relative to guppies. We show that this polymorphism is restricted to males and is maintained in natural populations of mainland South America. Using tests of female preference we show female *P*. *picta* are not more attracted to red males, despite preferences for red/orange in closely related species, such as *P*. *reticulata* and *P*. *parae*. Male color patterns in these closely related species are different from *P*. *picta* in that they occur in discrete patches and are frequently Y chromosome-linked. *P*. *reticulata* have an almost infinite number of male patterns, while *P*. *parae* males occur in discrete morphs. We show the red male polymorphism in *P*. *picta* extends continuously throughout the body and is not a Y-linked trait despite the theoretical prediction that sexually-selected characters should often be linked to the heterogametic sex chromosome. The presence/absence of red male coloration of *P*. *picta* described here makes this an ideal system for phylogenetic comparisons that could reveal the evolutionary forces maintaining mate choice and color polymorphisms in this speciose group.

## Introduction

Understanding the forces maintaining within species color polymorphisms has important implications across the fields of both ecology and evolutionary biology. Variation in coloration patterns are often associated with differences in behavior such as mating strategies (*e*.*g*. *Poecilia parae* [1,2], bluegill sunfish [3], side-blotched lizards [4]), microhabitat choice [5], and thermal regulation (*e*.*g*. land snails [6], and spittle bugs [7]). It has been suggested that these differences can lead to speciation across populations in parapatry, peripatry and even (although more controversially) sympatry (reviewed in [8]). Currently, most studies of color polymorphism focus on one species at a time, thus these studies lack the power of a phylogenetic approach when asking questions about the effects of color polymorphism on diversification. Wielding the power of phylogenetic analyses requires studying several species with well-characterized traits in a clade for which evolutionary relationships are well known [9].

The family Poeciliidae is a speciose group of small tropical freshwater fishes that has been well studied for the last 100 years and for which phylogenetic relationships are well known [10]. Sex specific color polymorphisms are especially well characterized for 2 species of Poeciliid, *Poecilia parae* [1], and *Poecilia reticulata* (reviewed in [11]). In both *P*. *reticulata* and *P*. *parae* males show color polymorphisms that play a large role in female mate choice (*P*. *reticulata*: [11,12], *P*. *parae* [13]) but attract unwanted attention from predators [13, 14, 15]. When there are sex-specific benefits to coloration, but costs to all carriers, this creates sexual conflict over the expression of color polymorphism [16]. This conflict is frequently resolved through linkage to heterogametic sex chromosomes [16, 17]. Male coloration in both *P*. *reticulata* and *P*. *parae* follow this pattern and have strong Y-linked components [1,18].

The guppy, *Poecilia reticulata*, is one of the premier models for studying the balance between sexual and natural selection (reviewed in [11, 19]). Males are much more colorful than females, and bear such an immense variety of colors and patterns that many populations have no two males that look identical [11]. Why this is so has been the subject of intense study for nearly one hundred years but these studies have largely lacked the strength of phylogenetic approaches.

*Poecilia parae*, a close relative of *P*. *reticulata*, also possesses extreme male color polymorphism [1] which has been shown to represent a balance between natural and sexual selection [2]. Interestingly, *P*. *parae* is often found in sympatry with guppies, yet male polymorphism is restricted to one of five discrete morphs: four color morphs (displaying body and tail stripes of red, yellow or blue, outlined in black, or only a multi-color tail stripe) and one juvenile-like morph [1], in contrast to the almost infinite number of male coloration patterns observed in guppies.

Another closely related species of Poeciliid is frequently found in sympatry with both *P*. *reticulata* and *P*. *parae* in northeast South America, including Guyana, Venezuela, and Suriname: *Poecilia picta*, also known as the swamp guppy. *P*. *picta and P*. *parae* are part of a clade that comprises the sister taxon to the guppy [20], within the subgenus *Lebistes sensu* Rosen and Bailey 1963 [21]. This sister taxon has been described as the genus *Micropoecilia* [22], but this is inconsistent with the standard taxonomy of the genus *Poecilia* as described by Rosen and Bailey [21]. Sexual color dimorphism has been described in *P*. *picta* from South America and Trinidad with males possessing an orange and black stripe on the top of their caudal fin and a spot on their dorsal fin [23]. Female mate choice appears to play an important role in *P*. *picta* as males display for females and females either accept or reject them [23, 24]. While orange plays a dramatic role in female mate choice in *P*. *reticulata* (reviewed in [11, 19]) conspicuous orange coloration on the caudal fin does not appear to play a role in female preference in *P*. *picta* [24].

**Fig 1:**
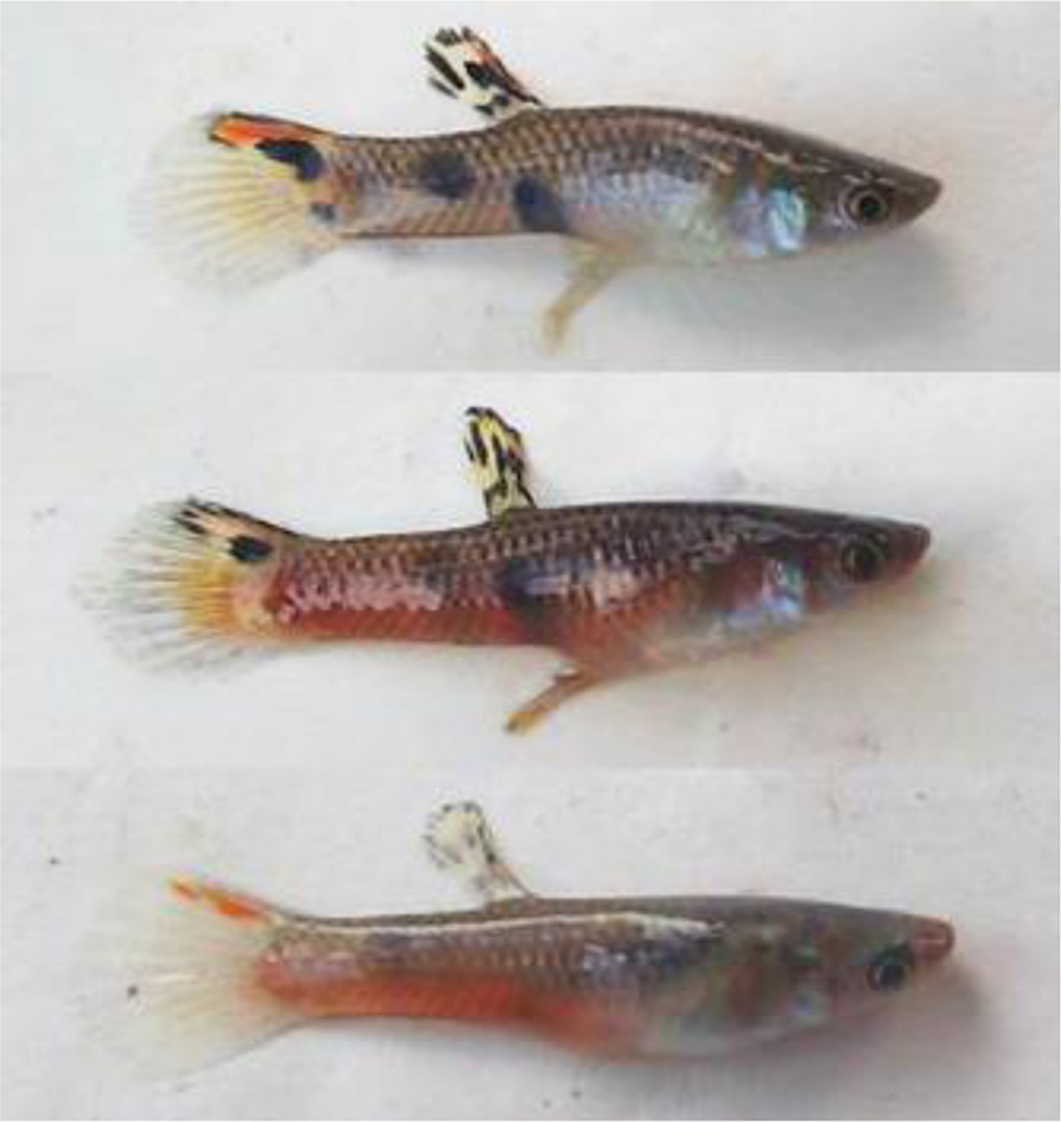
Standard (top), fully red (mid) and partially red (bottom) morph of *P*. *picta* from Georgetown, Guyana.

Here we report for the first time a striking red and a gold color sex-specific polymorphism in male *P*. *picta* from the mainland of South America that has persisted across a series of field surveys spanning 11 years. Previous studies of *P*. *picta* from the island of Trinidad suggest the absence of this color polymorphism in that island population. This red coloration can extend over most of the body of the male (Fig 1). In order to compare this to *P*. *reticulata* and *P*. *parae* male coloration, we investigate the inheritance pattern of the red morph through inbred lines maintained in the laboratory. Since natural selection via predation against red/orange morphs is countered by sexual selection via female mate choice in both of the close relatives, *P*. *reticulata* and *P*. *parae*, we used dichotomous choice tests to look for a female preference for the red color morph in *P*. *picta*.

## Materials and Methods

### Field surveys

*Poecilia picta* has been reported to occur in Guyana, French Guiana, Brazil and Trinidad and Tobago [21, 25, 26]. We have anecdotally observed the presence of the red morph within *P*. *picta* populations in Suriname, Guyana and Venezuela but not in Trinidad on collecting trips from 1983 to 2011. To quantify the frequency of the red morph, we searched for *P*. *picta* near the coast in ditches draining into rivers in four separate sets of field surveys in Guyana (June–August 1999; November 2000; January–February 2002; July 2010) and one field survey in Venezuela. In Guyana, *P*. *picta* were only found in mud-bottomed ditches of a variety of sizes which served as sewerage outlets. We considered different drainage systems into a river as different sampling sites. Adult males and females were captured with dip nets or a 3 x 1.5 m seine net, and male color pattern was scored. At the end of each sampling period, fish were either released or taken into captivity.

### Investigation of the genetic basis of the red color morph

Male and female *P*. *picta* were transported to Simon Fraser University, Canada from Georgetown, Guyana. We tested whether the loci underlying the red color morph conform to predictions of Y-linkage in 5 inbred lines founded by offspring of wild caught field-inseminated females. Two lines were started with red males and red male offspring were used as sires for the next generation, and the same for three lines of standard males. Full sibling crosses were set up in each generation for 2–4 generations per inbred line. In each generation, the number of red *vs*. standard males was scored at sexual maturity to assess whether the red phenotype is inherited as a Y-linked characteristic.

### Dichotomous female preference tests

Dichotomous choice tests were conducted in Georgetown, Guyana, using the same experimental apparatus and design as used for *P*. *parae* in Lindholm *et al*. [1]. In short, the social preferences of test females for red or standard morphs were assessed in two identical glass aquaria.

The test *P*. *picta* females were placed in a central compartment (23 x 30 cm) and could see two conspecific males in adjacent compartments (each 11 x 15 cm) at one end of the aquarium, and at the other end of the aquarium was a compartment (11 x 30 cm) which housed two conspecific females. Each aquarium contained 20 liters of rainwater and a tablespoon of sea salt. Tan gravel covered the bottom of the tanks. The walls of the tanks were covered with translucent waxed paper, except for the glass of the companion female compartment, through which the behavior of the test female and males was observed. Lighting was provided by natural daylight and an Aquari-lux FL-20 full-spectrum aquarium light suspended 37 cm above the water surface and centered between the two tanks, which were placed 11 cm apart.

At the start of a choice test, a test female, a red male and an opponent standard male were placed into their respective compartments. Red males used in the choice tests were slightly larger than standard males (mean difference of 0.3 ± 0.03 cm, paired t-test, t = 2.32, df = 24, *P* = 0.030) and showed strong red coloration. The red male was placed into the right compartment (with respect to the observer) in half of the trials. Opaque and clear glass partitions divided the two male compartments, and also the male compartments and the test female compartment. After a 10 minute acclimation period, the opaque partitions were removed, and male and female behavior was observed for 10 minutes. Removing the opaque partition between males was important because it allowed the males to see each other, which increased male courtship (as in guppies *P*. *reticulata* [27]). The opaque partitions were then replaced, and the males were switched between compartments. After another acclimation period, the opaque partitions were again removed and the fish were observed for an additional 10 minutes. The second observation period controlled for any side preferences of the female.

Fish behaviors were measured by the observer seated approximately 0.5 m away from the end of the tank housing the companion females. Event recorder software written by FB was used to record the number of seconds that the test female spent orientated toward each male against the glass of his compartment. The duration of attentiveness (defined as orientation to the female) was measured for each male. Trials were considered successful if the female was orientated towards at least one male in each of the two observation periods, if the female swam calmly in the tank, and if both males showed interest in the female. 39 trials were conducted, of which 25 were successful. Eighteen of these 25 females were tested within 48 hours of parturition, two were tested later in the reproductive cycle, and the remaining 5 females did not give birth in the laboratory. These trials tested female preference between 20 different red males paired with 18 different standard males. All fish used in the trials were wild-caught as adults. At the end of the study, animals were euthanized using MS-222. We used G*Power 3.1.9.2 for power analysis [28], whereas the base package of R 3.1.0 [29] was used for all other analyses.

### Ethics information

The Environmental Protection Agency of Guyana (permit #’s: 010301 BR 003, 120710 BR 135), Ministry of Agriculture of Guyana, Animal Husbandry and Fisheries of Suriname, Republica de Venezuela, Ministerio de Agricultura y Cría Servicio Autónomo de los Recursos Pesqueros y Acuícolas (permit #’s 0301, 0497) and the Trinidad Ministry of Housing and the Environment (permit # 001453) provided research and collecting permits. All fieldwork was done on publicly owned land and no protected species were sampled. Research was approved and animals were housed following animal protocol # 982B-06 granted by the Simon Fraser University Animal Care Committee.

## Results

### Distribution of red color morph in natural populations from northeast South America

**Fig 2:**
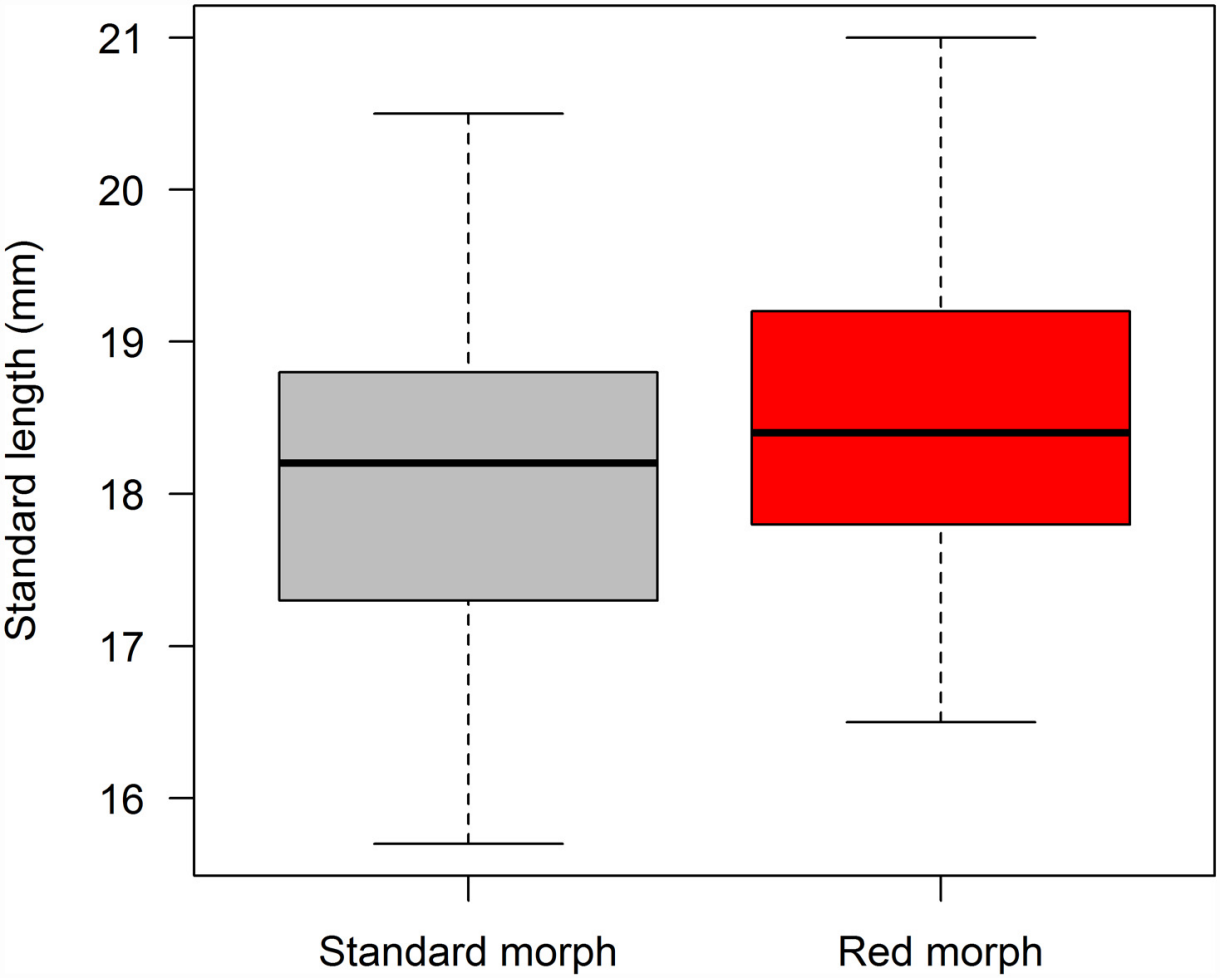
Box and whisker plot of standard length of standard and red males, indicating median value, upper and lower quartiles and minimum and maximum values.

Natural populations of *Poecilia picta* were observed on collecting trips to Venezuela, Guyana, Suriname and Trinidad, from 1983 through 2011. All *P*. *picta* males had black spots on their bodies and dorsal and tail fins ornamented with black and orange or yellow (Fig 1). The Venezuelan populations that were observed are either sympatric with the Cumana guppy in Cumana, or sympatric with standard guppies (*e*.*g*., Cano Pedernales and Tucupita in the Orinoco Delta, or Pozo Azufre, Estado Sucre) [30, 31]. The other populations that are sympatric with *P*. *reticulata* and that we observed to contain the red color morph are from Guyana (Georgetown, Demerara River, and New Amsterdam) and Suriname (Corentyne River population). Extent of red coloration varied, as 68.8% of males classified as red showed red color from the base of the tail to the snout (excluding black spots), as in Fig 1. The remainder were classified as partially red, showing red color on approximately half to one-third of the body; we combined all males showing any red coloration on the body when we report frequency of the red male morph in Table 1. Red males were larger in standard length than standard males (mean red males 18.6 ± 0.01 SE, n = 69, mean standard males 18.05 ± SE 0.01, n = 156, two sample t test, t = 3.43, df = 223, p = 0.0007, Fig 2;S1 Table). A few individuals from Guyana, 2 from the West Demerara, and 3 from West Berbice, showed full gold coloration instead of red.

**Table 1:** Survey of *P*. *picta* color morph frequency in Guyana and Venezuela.

		Coordinates						
Region	Site	N	W	Year	Red	Standard	Gold	% Red
**Guyana**								
Essequibo	Tuschen	6° 52.728	58° 20.991	2010	8	19	0	29.6
West Demerara	Pouderoyen	6° 47.797	58° 11.112	2002	12	75	1	13.6
				2010	0	24	0	0.0
				All	12	99	1	10.7
	Good Fortune	6° 47.148	58° 11.497	2002	21	95	0	18.1
	Patentia	6° 41.472	58° 11.858	2002	5	57	1	7.9
Georgetown	Seawall	6° 49.632	58° 07.346	1999	8	89	0	8.2
				2000	1	9	0	10.0
				2010	0	23	0	0.0
				All	9	121	0	6.9
	Botanical Gardens	6° 48.332	58° 08.720	1999	103	395	0	20.7
				2000	20	139	0	12.6
				2002	3	9	0	25.0
				2010	4	53	0	7.0
				All	130	596	0	12.6
	Turkeyen	6° 49.067	58° 06.764	2010	1	34	0	2.9
	All			All	140	751	0	15.7
East Demerara	Great Diamond	6° 43.312	58° 11.532	2010	4	90	0	4.3
	Timeri	6° 31.643	58° 15.062	2010	4	53	0	7.0
West Berbice	Rossignol	6° 16.347	57° 32.511	2002	5	16	3	20.8
Guyana	All			All	199	1180	5	14.4
**Venezuela**								
Orinoco Delta	Tucipita	9° 3.417	62° 2.983	199	2	12	0	14.3

Although some collections exhibited no red color morphs at some times, all regions observed on the mainland have exhibited the red color morph at some time point. We surveyed several of these populations intensively enough to report a frequency for the red color morph (N = 10 or more, Table 1). The frequency of the red color morph in the set of populations from Guyana compared to all other morphs ranged from 0% to 29.6%, with the proportion of red morph across all Guyanese collections being 199 of 1384, or 14.4%. This is similar to our largest collection from Venezuela, in which the red morph occurred in 2 of 14 adult males (14.3%). Overall, there is significant geographic variation in frequencies of the red morph (proportion test, *χ* ^2^ = 21.64, df = 8, p = 0.006).

Intriguingly, the red morph does not seem to occur on the South American “land bridge” island of Trinidad. We examined *Poecilia picta* from 3 populations spanning the island of Trinidad; Icacos Point in the far southwest, Tompire River in the northeast, and Caroni swamp in the western part of the Caroni drainage, and have never observed the red phenotype in any males. Reports of *P*. *picta* from Trinidad in the sexual selection literature also do not mention this red phenotype [32, 33, 34].

### Inheritance

In two to four generations of inbreeding, we found that lines started by males of the standard color morph never produced red morph male offspring, whereas lines started by red males produced both red and standard color offspring (Table 2). Male color patterns were stable, as we observed no males changing color morph during their lifespan. We can conclude from these breeding experiments so far that red coloration is not Y-linked, as opposed to many of the genes for male coloration in the guppy *P*. *reticulata* and male color morph in the closely related *Poecilia parae*.

**Table 2:** Inheritance results from inbred lines established by either red or standard morph males of *P*. *picta*.

Generation	Offspring Phenotype	Inbred Line				
		Red 1	Red 2	Standard 1	Standard 2	Standard 3
1	Red males	14	18	0	0	0
	Standard males	14	21	5	2	7
	Females	21	61	7	11	3
2	Red males	7	3	0	0	0
	Standard males	9	4	8	2	3
	Females	15	9	9	1	7
3	Red males	7	16	0	-	0
	Standard males	4	9	4	-	6
	Females	8	31	14	-	10
4	Red males	0	-	0	-	-
	Standard males	3	-	24	-	-
	Females	3	-	29	-	-

### Female Preference

**Fig 3:**
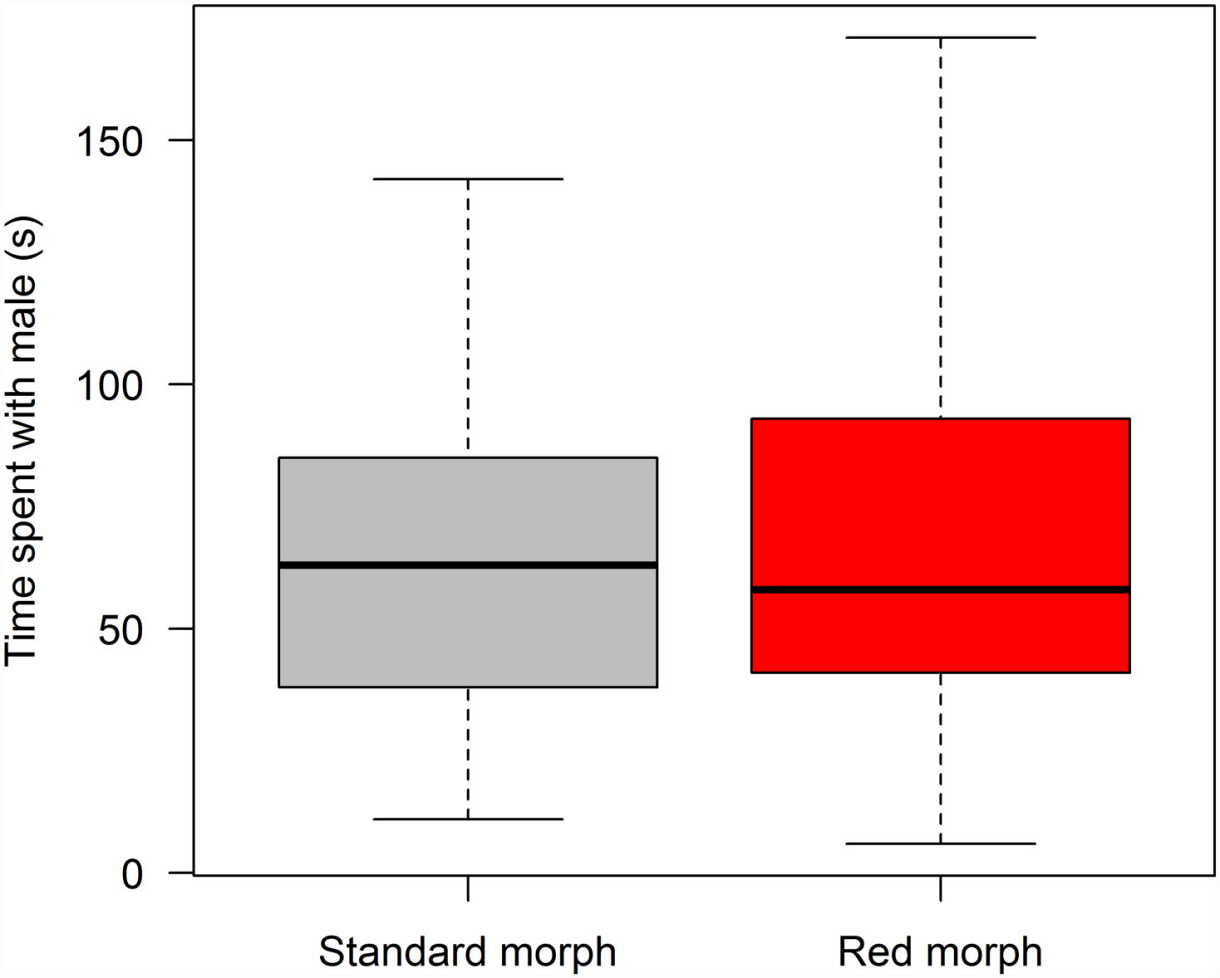
Duration of association with males in seconds (mean ± SE) in female preference tests.

Females showed no preference for either morph in paired choice tests with a red *vs*. standard male (Fig 3; paired Wilcoxon signed rank test, n = 25, V = 160, p = 0.96, S2 Table). Restricting the dataset to females within 1 day of parturition, as non-virgin female *P*. *reticulata* are most sexually active close to parturition [23], did not change this result (n = 18, V = 85.5, p = 1.00). Accounting for reuse of male pairs in four cases by averaging the results of the paired trials also did not change this result (paired Wilcoxon signed rank test, n = 21, V = 108, p = 0.96). There was no difference between display times of males within a trial (median for red males 961 s and standard males 960 s, Wilcoxon test V = 131.5, p = 0.41). Focal females spent significantly more time with conspecific females than with the test males (paired Wilcoxon signed rank test, n = 25, V = 24, p<0.001), suggesting that choosiness, the effort that females put in to assess males [35], may have been low. We assessed the power of this study to detect differences in preference function, the relative ranking of males [35]. For comparison we used a well-cited study [36] of female choice between male guppies from a high carotenoid treatment resulting in bright red/orange spots and low carotenoid treatment leading to dull red/orange spots using a similar apparatus to ours (albeit without using conspecific females). Using the software G*power 3.1 [28], the effect size in this study was calculated to be 1.048, with a preference for highly red/orange males. With 25 trials, our study has a 99% power to detect such a difference, 76.6% power to detect an effect size of 0.5, and 24.3% power to detect a small effect size of 0.2.

## Discussion

We describe for the first time a male specific color polymorphism in *Poecilia picta* that persists at a moderate frequency in nature. This polymorphism consists of a presence or absence of red, or in a few instances gold, coloration that runs through the body. Unlike in the closely related species *P*. *reticulata* and *P*. *parae* the red morph of *P*. *picta* does not appear to be preferred by females. Our work adds *P*. *picta* as another member of Poeciliidae to possess sex specific male color polymorphism, but sets it apart in terms of the forces maintaining the polymorphism and its inheritance pattern. We first discuss the potential mechanisms that could explain the presence/absence of the red/gold morph polymorphism across populations. We then discuss the unique characteristics of the color polymorphism in *P*. *picta*, and suggest ways in which studies utilizing this male color-polymorphism will further the fields of ecology and evolutionary biology.

Red male coloration is common among fishes and red male coloration plays a large role in the life history of species closely related to *P*. *picta* including the sister species *P*. *reticulata* [11, 19] and *P*. *parae* [1, 2]. Red coloration is well known to cause individuals in shallow freshwater habitats to be more conspicuous to predators leading to strong natural selection against red phenotypes [37]. Indeed, both *P*. *reticulata* and *P*. *parae* are well known to experience strong natural selection acting against red/orange male coloration (*P*. *reticulata* [11, 19, 37, 38], *P*. *parae* [2, 39]). In terms of evolutionary dynamics, we consider orange and red coloration functionally equivalent, in that their perception is controlled by the long-wave sensitive family of opsins and the frequencies in the spectrum are very close. Both *P*. *reticulata* and *P*. *parae* are roughly the same size and shape and occupy similar ecological niches as *P*. *picta* [21, 23], especially in the Guyanese and Venezuelan populations where the 3 species occur in sympatry and where the red morph is found in similar frequencies. Therefore it would be expected that *P*. *picta* also experiences strong natural selection acting against red male coloration. In both *P*. *reticulata* and *P*. *parae* the adverse effects of natural selection are opposed, and even overcome, by sexual selection in which females prefer to mate with red/orange males (*P*. *reticulata* [40, 41], *P*. *parae* [2, 39]). The results of our study show that female *P*. *picta* are not strongly attracted to red morph males, yet the red morph is maintained both across populations and within populations across time. This suggests that natural selection acting against red male morphs may be opposed by another form of selection in *P*. *picta* selecting for the red male morph. Indeed, we show the frequency of red male phenotype varies across populations and within populations over time. Ultimately this distribution of morph frequencies would represent a balance among opposing selection pressures and migration among populations.

Over the last 80 years, populations of *P*. *picta* in Trinidad and Tobago have been the focus of studies on sexual selection [42], mate recognition [32, 33], heterospecific insemination [34], life history [43], parasite resistance [44] and ecological niches [45]. Despite the extensive body of work on Trinidadian populations of *P*. *picta* the red male morph has never been noted in Trinidad and Tobago. This supports the idea that there are forces working against the red male phenotype. There remain two likely processes that could explain the maintenance of the red male phenotype in *P*. *picta*: (1) intrasexual selection or (2) correlated characters. We explore these possible processes below.

Sexual selection can occur either intersexually (usually due to mate choice by females for attractive males) or intrasexually (usually due to competition between males) [46]. Our results reveal intersexual selection is not acting strongly on the red male phenotype in *P*. *picta*; however it is possible that male-male competition could result in the red males having higher reproductive success than the non-red males. If red males indeed have higher fitness than non-red males it would at first seem surprising that there are no red males in the *P*. *picta* populations on the islands of Trinidad and Tobago. However, Kaneshiro proposes that male sexual signals can be lost in island populations, which could explain the lack of red on the islands of Trinidad and Tobago [47, 48]. Alternatively, the relative fitness gain through male-male competition could be density dependent as seen in a wide range of taxa and models (reviewed in [49]). If this is the case then variation in density across populations could lead to a loss of red morph when the relative fitness gained through male-male competition no longer exceeded natural selection acting against the red phenotype.

While intrasexual selection could explain the maintenance of the red male polymorphism in *P*. *picta*, another possible process could be at play: the red phenotype may be correlated to a character that is not involved in sexual selection. A growing body of literature across a wide range of taxa shows that color polymorphisms can be maintained as correlated characters (reviewed in [50]). If this is the case then the variation in red male morph frequency across populations could be due to differences in selective pressure for the unknown character to which the red morph is linked.

Understanding the patterns, implications, and ubiquity of mechanisms underlying the maintenance of genetic polymorphisms in nature has been a long-standing question in the fields of ecology and evolution, and the forces maintaining these polymorphisms were succinctly summarized in Ford’s seminal book in 1965 [51]. Color polymorphisms have been shown to be capable of maintaining genetic diversity within individual species across a wide range of taxa (reviewed in [8]). Yet these taxa are largely disparate and their evolutionary relationships uncertain, making phylogenetic approaches difficult. Poeciliidae is a well studied family with species known to maintain high genetic diversity [52], often through frequency dependent selection based on color polymorphisms [2, 12]. By adding *P*. *picta* as another member of Poeciliidae with male color polymorphism, future work will be able to wield the power of phylogenetic analyses to answer long standing questions such as ‘does color polymorphism coincide with faster rates of speciation?’.

The lack of female preference for red male coloration in *P*. *picta* is perhaps surprising as guppies (*P*. *reticulata*) and *P*. *parae*, close relatives, exhibit strong female preferences for red/orange male coloration. It should be noted that males and females were not allowed to interact with each other in the choice tests in this study, and courtship in *P*. *picta* involves the male circling the head of the female; therefore the lack of preference in our tests could be an artifact of the study design. However, a separate test for female preference for the orange stripe on the caudal fin of *P*. *picta* males, which did allow for male/female interactions, did not show preference for orange signals [24], supporting the lack of female preference for orange and red coloration in *P*. *picta*. It has been proposed that female attraction to red/orange male coloration arose due to a pre-existing bias in guppies [53]. If such a pre-existing bias does indeed underlie female mate choice for red in guppies then closely related species could also demonstrate such female preferences [24]. *P*. *parae* females are also attracted to red conspecific males [1]. However, *P*. *picta* is one of the closest relatives to guppies [20, 54], and yet does not demonstrate female preferences for red male coloration. This lends support to alternative hypotheses of female preference for red coloration in guppies, such as sensory exploitation [55].

Despite theoretical predictions that suggest the inheritance of a sex specific coloration should be linked to the heterogametic sex, we found the inheritance pattern of the red morph in *P*. *picta* not to be Y-linked. This contrasts with multiple studies of Poeciliids, reviewed in [17], that show that not only most color patterns, but also male size and courtship patterns, tend to be linked to the heterogametic sex chromosome. The red morph in *P*. *picta* is also distinct in that it does not appear as a discrete color patch (Fig 1), as do most of the male coloration patterns in Poeciliids. This difference in appearance and inheritance suggests that this red male color polymorphism has evolved independently of the other types of red/orange coloration in guppies and close relatives.

The other phylogenetic contrast that our results for *P*. *picta* sets up involves the distribution of male coloration patterns in guppies and its sister clade [17]. Two species in the sister clade to the guppy have been studied, *P*. *parae* and now *P*. *picta*, and they both exhibit discrete male morphs: 5 in *P*. *parae*, and the red, standard and a rare gold morph in *P*. *picta*. The inheritance of the 5 morphs in *P*. *parae* is controlled by a single Y-linked locus, while the inheritance of the red morph in *P*. *picta* is not Y-linked. Further work will be needed to resolve the details of the inheritance pattern. This contrasts very clearly with *P*. *reticulata*, in which the male coloration patterns are controlled by as many as 30–40 loci, which produce highly polymorphic patterns such that almost every male within a population can be distinct [17]. This contrast could be very informative for the forces initiating and maintaining color polymorphisms in nature.

In summary, our work shows there to be a male specific color polymorphism that occurs in natural populations of *P*. *picta* that is not Y-linked and does not seem to play a role in female mate choice. It remains for further study to determine the precise mechanisms responsible for maintaining the red color polymorphism in nature. However, the presence of a non Y-linked male color polymorphism should prove useful in further studies investigating color polymorphism, sexual conflict, genetic diversity, and even female mate preference of closely related species.

## Supporting Information

S1 Table Standard lengths of standard and red color morphs of *Poecilia picta*. (TXT)

S2 Table Female preference test data. (TXT)
